# The journal's first year of publication and the challenges ahead

**DOI:** 10.1186/2045-4015-1-52

**Published:** 2012-12-21

**Authors:** Avi Israeli, Bruce Rosen

**Affiliations:** 1 Hebrew University-Hadassah Faculty of Medicine, POB 12000, Jerusalem, 91120, Israel; 2 Ministry of Health, Ben Tabai 2, Jerusalem, 93591, Israel; 3 Myers-JDC-Brookdale Institute, POB 3886, Jerusalem, 91037, Israel

## Abstract

*The Israel Journal of Health Policy Research (IJHPR)* seeks to promote intensive intellectual interactions among scholars and practitioners from Israel and other countries regarding all aspects of health policy and health care, with a special focus on Israel. During 2012, its first year of operation, the journal succeeded in publishing an impressive volume of policy-relevant articles by a remarkably diverse set of authors. The journal's success to date would not have been possible without the vital contributions of the editorial board, the authors, the reviewers, the readers, BioMed Central (the journal's publisher), and the Israel National Institute for Health Policy (the journal's sponsor). The challenges ahead include promoting greater reader involvement, and enhancing the journal's policy and educational impact.

## 

Now that the *IJHPR* has completed its first year of publication, we wanted to share with you our perceptions of the journal’s accomplishments to date and of the challenges ahead.

The *Israel Journal of Health Policy Research (IJHPR)* seeks to promote intensive intellectual interactions among scholars and practitioners from Israel and other countries regarding all aspects of health policy and health care, with a special focus on Israel. The ultimate aim of these intellectual interactions is to contribute to the development of health policy in Israel and around the world [1].

As such, the *IJHPR* seeks to fill an important gap in the creation and dissemination of new knowledge, which can contribute to both understanding and action. The *IJHPR* employs an on-line, open access dissemination model to reach as wide an audience as possible and to facilitate interactive reader involvement. A thorough peer-review process, typically involving two reviewers from Israel and two from abroad for each manuscript, ensures both scientific rigor and policy relevance.

To further its objectives, the *IJHPR* has consistently published at least four articles every month since its January 2012 launch, for a total of 52 articles. It is exciting that the articles have covered a broad range of topics, from egg cell donations to health care manpower, and from patient safety to obesity reduction. We have also been pleased to publish articles related to several aspects of Israeli health care that are of particular interest internationally, including health information technology, quality monitoring and improvement, emergency preparedness, deliberate priority setting, and managed care. In the year ahead, we hope to develop some of these into article collections.

Perhaps in part because of the broad range of topics, perhaps in part because of the open access policy, the journal's articles also seem to be reaching a broad audience. By mid-December, 4 of the 52 articles had been accessed over 2,000 times and 13 of them (more than a quarter) had been accessed over 1,000 times – including several that were only published over the last few months. In total, there have been over 40,000 article accesses from 132 countries (mostly from Israel, the US and the UK). These access statistics are considered good for a first year journal, and the experience of other BioMed Central journals suggests a promising future. We have clearly struck a chord among many readers. At the same time, we would welcome suggestions for how to reach an even wider audience and how to promote more active reader involvement.

Our sense is that the articles have been consistently solid, and occasionally brilliant. One of our greatest sources of joy as editors has been seeing several promising submissions, which initially needed further development, mature into first-rate manuscripts as a result of detailed, constructive input from peer reviewers and effective incorporation of that input by the authors. Many of our authors have told us that they have been impressed by the seriousness and the helpfulness of the reviews they have received. They also express a great deal of appreciation for the work of the journal's language editor.

We believe that publishing commentaries from internationally-recognized scholars has worked well. The commentaries provide our readers with additional insights on the issues raised by the main articles and a deeper understanding of their international significance. The authors of the main articles and the commentaries also benefit from the connections made between them, which in some cases have been continuing. We also know that the commentaries contribute greatly to the journal's status as a prestigious international journal.

We are gratified that, to date, we have received submissions from some of Israel's leading scholars as well as quite a few promising young researchers. Moreover, virtually all of Israel's universities and health services research centers are represented among the *IJHPR*'s authors. It has also been gratifying that several authors have now submitted their second or even third *IJHPR* manuscript, suggesting that their initial experiences were positive. We continue to be eager to see more repeat authors, while at the same time we are looking to editorial board members to help us broaden our circle of first-time authors.

Several of the articles published to date have been authored jointly by Israeli scholars and scholars from abroad. Such collaborations can greatly enrich the analysis and enhance its relevance to policymakers in many countries. We hope to see more collaborative submissions in the months ahead.

We understand that the articles have started to make their way into course curricula. We would welcome your feedback on the extent to which this is happening and what we might do to promote the use of the *IJHPR* for pedagogical purposes.

Several of the articles have already fed into the policy development process in Israel and some have received widespread media coverage. An important challenge for the *IJHPR* in 2013 will be to find ways to become even more integrated into the policy process.

We want to take this opportunity to thank the National Institute, the journal's sponsor, for its ongoing financial, intellectual, and moral support. We also want to thank the members of the editorial board for their help in soliciting manuscripts and charting the journal's overall direction. We are also greatly appreciative of the strong vote of confidence that the journal has received from the Council of Deans of Israeli Medical Schools.

This is also a good opportunity to thank our colleagues at BioMed Central, our publisher, for its ongoing assistance and support. Increasingly, leading foundations, funders, and scientific societies around the world (such as the NIH and the Wellcome Trust) are embracing the open access approach [2,3] and BioMed Central is among the leaders of the Open Access movement.

Many thanks to the over 100 scholars from around the world who have participated in the review process and whose input has contributed immensely to the quality of the articles published. Without their contribution the journal would not be what it is today. A full list of *IJHPR* reviewers to date will be appearing in a forthcoming *IJHPR* article.

Finally, a big thanks to the dozens of authors who have submitted their manuscripts to the journal. We are constantly mindful of the fact that other publication outlets are available to you and that, without your belief in the *IJHPR*, there would be no journal. We hope that those of you whose articles have been published in the *IJHPR* have had a good experience with us and we welcome your feedback. We also hope that those whose submissions have not made it through the process will not be discouraged and will consider submitting other manuscripts to us in the future.

We conclude by wishing all of us many more years of fruitful cooperation. We are confident that our readers will find ways to use the articles for the benefit of population health in Israel and around the world.
